# Expression Profile of SARS-CoV-2 Host Receptors in Human Pancreatic Islets Revealed Upregulation of *ACE2* in Diabetic Donors

**DOI:** 10.3390/biology9080215

**Published:** 2020-08-07

**Authors:** Jalal Taneera, Waseem El-Huneidi, Mawieh Hamad, Abdul Khader Mohammed, Esraa Elaraby, Mahmood Yaseen Hachim

**Affiliations:** 1 Sharjah Institute for Medical Research, University of Sharjah, Sharjah 27272, UAE; amohammed@sharjah.ac.ae (A.K.M.); esrahmed@live.com (E.E.); 2 Department of Basic Medical Sciences, College of Medicine, University of Sharjah, Sharjah 27272, UAE; welhuneidi@sharjah.ac.ae; 3 College of Health Sciences, University of Sharjah, Sharjah 27272, UAE; mabdelhaq@sharjah.ac.ae; 4 College of Medicine, Mohammed Bin Rashid University of Medicine and Health Sciences, Dubai 505055, UAE; mahmood.almashhadani@mbru.ac.ae

**Keywords:** COVID-19, Diabetes, human islets, *ACE2*, *ADAM17*, *TMPRSS2*

## Abstract

Cellular entry of SARS-CoV-2 is thought to occur through the binding of viral spike S1 protein to *ACE2*. The entry process involves priming of the S protein by *TMPRSS2* and *ADAM17*, which collectively mediate the binding and promote ACE2 shedding. In this study, microarray and RNA-sequencing (RNA-seq) expression data were utilized to profile the expression pattern of *ACE2*, *ADAM17*, and *TMPRSS2* in type 2 diabetic (T2D) and non-diabetic human pancreatic islets. Our data show that pancreatic islets express all three receptors irrespective of diabetes status. The expression of *ACE2* was significantly increased in diabetic/hyperglycemic islets compared to non-diabetic/normoglycemic. Islets from female donors showed higher *ACE2* expression compared to males; the expression of *ADAM17* and *TMPRSS2* was not affected by gender. The expression of the three receptors was statistically similar in young (≤40 years old) versus old (≥60 years old) donors. Obese (BMI > 30) donors have significantly higher expression levels of *ADAM17* and *TMPRSS2* relative to those from non-obese donors (BMI < 25). *TMPRSS2* expression correlated positively with HbA1c and negatively with age, while *ADAM17* and *TMPRSS2* correlated positively with BMI. The expression of the three receptors was statistically similar in muscle and subcutaneous adipose tissues obtained from diabetic and nondiabetic donors. Lastly, *ACE2* expression was higher in sorted pancreatic β-cell relative to other endocrine cells. In conclusion, *ACE2* expression is increased in diabetic human islets. More studies are required to investigate whether variations of *ACE2* expression could explain the severity of COVID-19 infection-related symptoms between diabetics and non-diabetic patients.

## 1. Introduction

In March 2020, the World Health Organization declared that coronavirus disease 2019 (COVID-19) or severe acute respiratory syndrome coronavirus (SARS-CoV-2) was a global pandemic [1]. The elderly and patients with diabetes mellitus are at higher risk of COVID-19 and developing severe symptoms that are often fatal [2]. The rate of survival in diabetics with COVID-19 is significantly reduced (22–31%) relative to non-diabetic counterparts [3]. Previous work has attributed this to compromised immunity in diabetics; innate and humoral adaptive responses in particular [4]. Additionally, transient hyperglycemia has been documented in 50% of COVID-19 patients in Wuhan, which could be due to SARS-CoV-19 binding to host receptors in pancreatic islets [5]. Indeed, transient impairment of pancreatic islet cell function was reported in 2003 with severe Acute Respiratory Syndrome (SARS) infection [6]. It has been reported that diabetic patients with COVID-19 continued to show uncontrolled hyperglycemic even when blood glucose management strategies were applied [7]. Such uncontrolled hyperglycemic levels will expose those patients to secondary infections along with higher mortality risks.

The mode of cellular entry of the SARS-CoV-2 is thought to occur through the binding of viral spike S1 protein to the angiotensin-converting enzyme 2 (*ACE2*) on the surface of alveolar epithelial cells and the subsequent endocytosis and translocation of the complex into the cytoplasm [8,9]. The entry process also involves the priming of the S protein by the host serine protease protein (*TMPRSS2*) and a disintegrin and metalloproteinase 17 (*ADAM17*), which together mediate SARS-CoV-2 binding to *ACE2* on target cells and promote ACE2 shedding from endothelial cells [10]. Stable expression of *ACE2* has been shown to protect against COVID-19 infection, especially in lung injury [11].

ACE2 is expressed on different types of cells of kidney, heart, vasculature, gastrointestinal tract, smooth muscle, liver, and pancreatic tissues [12,13]. The receptor plays a crucial role in anti-oxidation and anti-inflammation as it degrades angiotensin-II and—to a lesser extent—angiotensin I into smaller peptides known as angiotensin 1–7 [14]. The fact that this process is likely to be compromised in diabetics makes them more vulnerable to increased risk of severe lung injury and acute respiratory distress syndrome (ARDS) [15]. Although decreased expression of *ACE2* could be a helpful strategy to fight the infection, *ACE2* has been shown to have a protective effect against virus-induced lung injury by increasing the production of the vasodilator angiotensin 1–7 [14].

*ACE2* expression has been reported on humans [6] and rodent pancreatic islets [16] and its role in diabetes and β-cell function is well recognized [17,18]. *ACE2* deficient mice exhibit decreased glucose tolerance and reduced first-phase insulin secretion [19]. Moreover, *ACE2* gene therapy in the *db/db* mice resulted in improved fasting blood glucose levels, glucose tolerance along with increased first-phase insulin secretion and β-cell proliferation [20]. However, the exact role of *ACE2* in COVID-19 infection in diabetics requires further consideration. Additionally, the expression pattern of *TMPRSS2* and *ADMA17* needs to be investigated. In this study, we utilized our human islets microarray and RNA-seq expression data to profile the expression of these three receptors in diabetic and non-diabetic samples and correlate their expression with other phenotypes of significance in diabetes including age, sex, BMI and HbA1c levels.

## 2. Materials and Methods

### 2.1. Microarray Gene Expression from Human Pancreatic Islets

To profile the expression of the three receptors in human pancreatic islets, we retrieved our previous microarray gene expression data (Publicly available database; GEO, accession number: GSE41762). The microarrays (GeneChip^®^ Human Gene 1.0 ST, Waltham, MA, USA) were performed using the Affymetrix standard protocol as previously described [21]. The raw Affymetrix data were normalized with Robust Multi-array Analysis method using the ligo package from BioConductor. As previously described [21], pancreatic islets were isolated from 76 cadaver donors, of which 67 were non-diabetic and 9 were patients with T2D. Characteristics of human islets donors are showed in Table 1.

### 2.2. RNA-Seq Data for Human Pancreatic Islets

RNA-seq data (GSE50398) were obtained from 89 cadaver donors (non-diabetic HbA1c < 6%, *n =* 66 and T2D/hyperglycemic HbA1c ≥ 6.3%, *n =* 12). Data normalization was processed using a trimmed mean of M-values and presented as Fragments/Kilobase of Exon Per Million Fragments Mapped (FPKM) or transformed into log2 counts per million using the voom-function (edgeR/limma R-packages) as previously described [22]. Pancreatic islets were isolated from 89 cadaver donors, of which 75 were non-diabetic (30 females, 45 males, age 62 ± 10, BMI 25.4 ± 2.8, and HbA1c% 5.5 ± 0.4) and 12 were patients with T2D (6 females, 6 males, age 65 ± 11, BMI 29.4 ± 3.2, and HbA1c% 7.3 ± 1.0).

### 2.3. Receptor Expression in Other Tissues

To profile the gene expression of the three receptors in muscle and subcutaneous adipose tissues from diabetic versus healthy controls, we explored datasets (GSE40234 and GSE29221) from A Gene Atlas of Type 2 Diabetes Mellitus Associated Complex Disorders (T2DiACoD) using the online tool (http://t2diacod.igib.res.in/tissue_expr.php). The normalized gene expression of the three genes was extracted from each dataset using the GEO2R tool (https://www.ncbi.nlm.nih.gov/geo/geo2r/). For the GSE40234 dataset [23], we explored sixty-two participants that were well matched for age, gender, BMI, and percent fat, but were different for insulin sensitivity. Participants were aged 20 years to 55 years, with a body mass index (BMI) between 19 and 42 kg/m^2^, and biopsies were obtained during a fasting state. For the GSE29221 dataset, biopsy samples of the skeletal muscle of three T2D male patients and three non-diabetic patients were used for gene expression profiling. The average age of the patients was 58 years (range 37 to 85 years). The levels of glycated hemoglobin (%HbA1c) were 5.75 ± 0.33 and 9.44 ± 0.82 in nondiabetic and diabetic patients, respectively, with a significant difference between the two groups (*p* = 0.003). The BMI were 24.48 ± 1.2 and 25.00 ± 1.81 of nondiabetic and diabetic patients, respectively, with an insignificant difference between the two groups (*p* = 0.81) [24].

### 2.4. Statistical Analysis

Spearman’s correlation test was used to assess the degree of correlation between gene expression and phenotype (age, sex, etc.). Non-parametric unpaired t-test (Mann–Whitney test) was used to assess significance levels in differential gene expression data. Statistical analyses were performed using GraphPad Prism (version 8.0.0 for Windows, GraphPad Software, San Diego, CA, USA, www.graphpad.com). Differences were considered significant at *p* < 0.05.

## 3. Results

**Figure 1:**
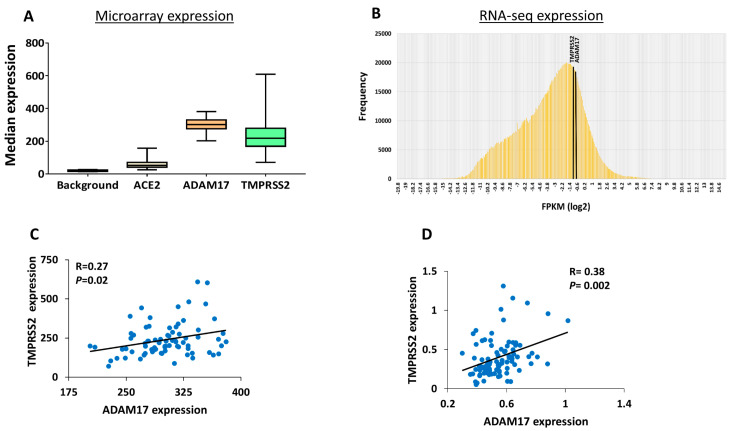
Gene expression profile of ACE2, ADAM17 and TMPRSS2 in human pancreatic islets. (**A**) Microarray expression of ACE2, ADMA17 and TMPRSS2 in non-diabetic (*n =* 64) donors. Background signal levels of all negative control probe sets were calculated based on Human Gene 1.0 ST. (**B**) RNA-seq histogram expression frequency (FPKMs) of ADMA17 and TMPRSS2 in non-diabetic human pancreatic islets (*n =* 64). (**C**) Microarray expression correlation of ADAM17 with TMPRSS2 (*n =* 75). (**D**) RNA-seq expression correlation of ADAM17 with TMPRSS2 (*n =* 89). *p*- and *R*-values are shown as per the respective figure.

A complete description of the expression levels of *ACE2*, *ADMA17* and *TMPRSS2* in normal non-diabetic human pancreatic islets is still lacking. Therefore, we analyzed the microarray expression of the three receptors in human islets. All the studied receptors were found to be expressed in/on human pancreatic islets and their expression was above background control signal, which was calculated based on the mean values of all negative control probesets on the array [25,26] (Figure 1A). *ADAM17* and *TMPRSS2* showed a significantly higher expression level (*p* < 0.01) compared to *ACE2* in pancreatic islets (Figure 1A). RNA-seq revealed a relatively high expression of *TMPRSS2* and *ADAM17* in human islets (Figure 1B). We could not analyze the expression of the *ACE2* receptor as it was not aligned in the RNA-sequencing data. Microarray expression analysis of the three receptors revealed a positive correlation between *ADAM17* with that of *TMPRSS2* (Figure 1C). Similar results were obtained from RNA-seq (Figure 1D). No correlations were observed for *ACE2* with *ADAM17* or *TMPRSS2*.

**Figure 2:**
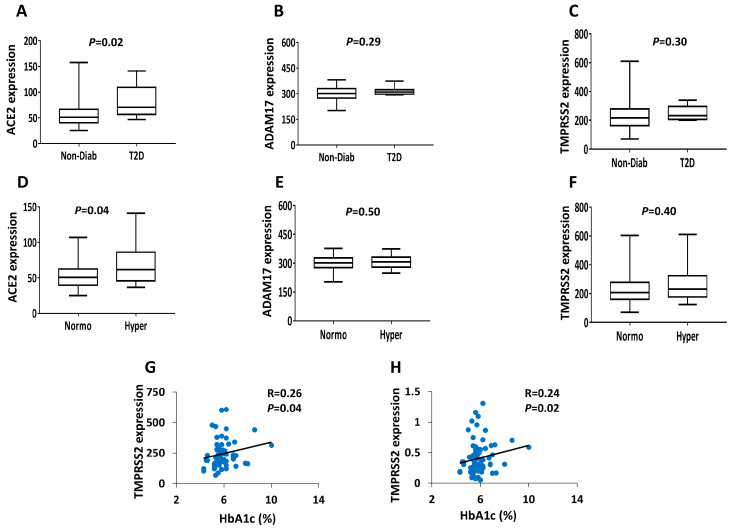
Impact of diabetes status and hyperglycemia on the expression of *ACE2*, *ADAM17* and *TMPRSS2* in human pancreatic islets. (A–C) Microarray expression of *ACE2* (**A**), *ADMA17* (**B**) and *TMPRSS2* (**C**) in diabetic (*n* = 8) and non-diabetic (*n* = 64) donors. (D–F) Microarray expression of *ACE2* (**D**), *ADMA17* (**E**) and *TMPRSS2* (**F**) in hyperglycemic (*n* = 23; HbA_1c_ ≥ 6%) versus normoglycemic (*n* = 44; HbA_1c_ < 6%) donors. (**G**) Microarray expression correlation of *TMPRSS2* with HbA1c level (*n* = 63). (**H**) RNA-seq expression correlation of *TMPRSS2* with HbA1c (*n* = 77). *p*- and *R*-values are shown as per the respective figure.

Next, we compared microarray expression data for *ACE2*, *ADAM17* and *TMPRSS2* between diabetic versus non-diabetic donors as well as between hyperglycemic (HbA1c ≥ 6%) versus normoglycemic (HbA1c < 6%) donors. As shown in Figure 2, the expression of *ACE2* was significantly (*p* ≤ 0.05) upregulated in diabetic or hyperglycemic islets compared to non-diabetic or normoglycemic (Figure 2A,D). No significant differences were evident regarding the expression of *ADAM17* and *TMPRSS2* in relation to diabetes status and/or glycemic levels (Figure 2B–F). Likewise, we could not observe any differential expression of *ADAM17* and *TMPRSS2* in human islets using RNA-seq data (Not shown). Correlation analysis from microarray and RNA-seq expression data showed a positive association between *TMPRSS2* and HbA1c (Figure 2G,H); no correlation was evident regarding the expression of *ACE2* or *ADAM17* with HbA1c levels. No associations were detected between the microarray expression of the three genes and measured insulin secretion of human islets (not shown).

**Figure 3:**
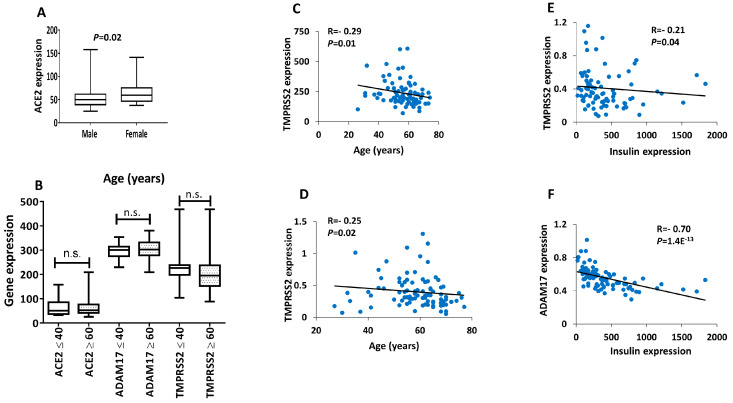
Impact of gender and age on expression levels of *ACE2*, *ADAM17* and *TMPRSS2* in human islets. (**A**) Microarray expression of *ACE2* obtained from male donors (*n* = 45) versus females (*n* = 30). (**B**) Microarray expression of *ACE2*, *ADMA17* and *TMPRSS2* from donors ≤ 40 years old (*n* = 6) versus ≥ 60 years (*n* = 31). (**C**) Microarray expression correlation of *TMPRSS2* with age (*n* = 76). (**D**) RNA-seq expression correlation of *TMPRSS2* with age (*n* = 77). RNA-seq expression correlation of TMPRSS2 (**E**) or ADAM17 (**F**) with insulin gene expression (*n* = 85). N.S.; not significant. *p*- and *R*-values are shown as per the respective figure.

Interestingly, microarray expression of *ACE2* showed higher expression levels in islets obtained from females as compared to males (Figure 3A). The expression of *ADMA17* or *TMPRSS2* was unchanged on gender-based on microarray or RNS-seq expression data (Not shown). To address whether age has any bearing on the expression of *ACE2*, *ADAM17* and/or *TMPRSS2*, we stratified the donors’ age into young (≤40 years old) and old (≥60 years old). None of the three receptors showed any differential expression relative to age in the microarray dataset (Figure 3B). Similar results were obtained from RNA-seq dataset (not shown). Of the three studied receptors, *TMPRSS2* was correlated inversely with age in both microarray and RNA-seq datasets (Figure 3C,D). *ACE2* and *ADAM17* showed no correlations. Co-expression analysis of ADAM17 and TMPRSS2 with the gene expression of insulin using RNA-seq data showed that both of the genes are inversely correlated with insulin expression (*R*^2^ = −0.70; *p* = 1.4 × 10^−13^ and *R*^2^ = −0.21; *p* = 0.04, respectively) (Figure 3E,F).

**Figure 4:**
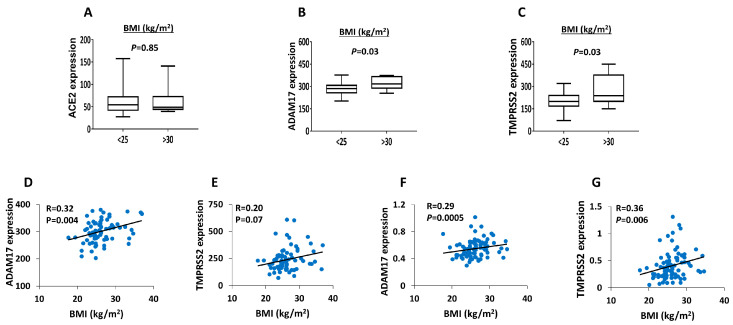
Impact of BMI on expression levels of *ACE2*, *ADAM17* and *TMPRSS2* in human islets. (**A**–**C**) Microarray expression of *ACE2* (**A**), *ADMA17* (**B**) and *TMPRSS2* (**C**) obtained from donors with BMI < 25 (*n* = 34) versus BMI > 30 (*n* = 10). (**D**–**E**) Microarray expression correlation of *ADAM17* (**D**) and *TMPRSS2* (**E**) with BMI (*n* = 76). (F–G) RNA-seq expression correlation of *ADAM17* (**F**) and *TMPRSS2* (**G**) with BMI (*n* = 89). *p*-and *R*-values are shown as per the respective figure.

Likewise, we stratified the donors into normal (BMI < 25) and obese (BMI > 30) as a means of testing the effect of obesity on the expression of these receptors. The expression of *ADAM17* and *TMPRSS2* was significantly (*p* < 0.05) higher in obese donors compared to non-obese ones (Figure 4B,C). *ACE2* showed no correlation (Figure 4A). Additionally, using the microarray and RNA-seq datasets, we found that *ADAM17* and *TMPRSS2* correlated positively with BMI values (Figure 4D–G).

To test whether the expression of these receptors is influenced by pancreatic cell type, we analyzed the expression of *ACE2*, *ADMA17,* and *TMPRSS2* using RNA-seq data from sorted endocrine and exocrine cells [27]. As shown in Table 2, *ACE2* expression was relatively higher in β-cells (0.27 normalized expression values) as compared with α (0.18) or exocrine cells (0.21). *ADMA17* exhibited similar expression levels in β and α cells (1.5 and 1.4, respectively). *ADMA17* was highly expressed (3.1) and *TMPRSS2* was very highly expressed in exocrine cells (5.1) as compared with that in α (1.02) or β cells (0.55).

**Figure 5:**
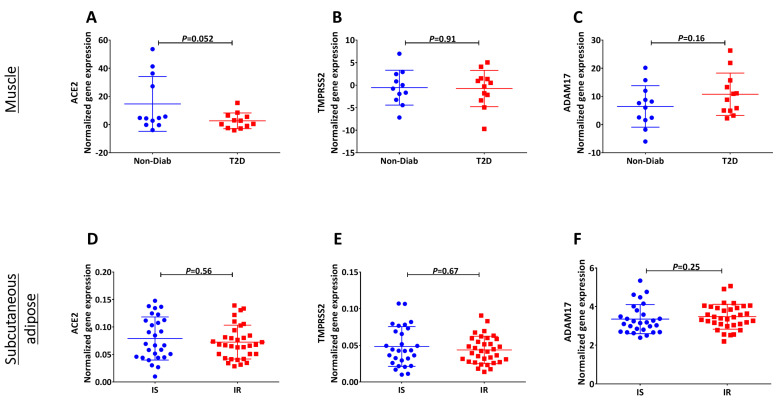
Microarray gene expression of *ACE2*, *ADAM17* and *TMPRSS2* in diabetic tissues. (**A**–**C**) Mean expression of *ACE2* (**A**), *ADAM17* (**B**) and *TMPRSS2* (**C**) in muscle tissues obtained from diabetic and healthy donors. (**D**–**F**) Mean expression of *ACE2* (**D**), *ADAM17* (**E**) and *TMPRSS2* (**F**) in subcutaneous adipose tissues obtained from diabetic and normal donors.

Lastly, using the Gene Atlas of Type 2 Diabetes Mellitus Associated Complex Disorders (T2DiACoD), we investigated the impact of diabetes status on the expression of the three receptors in muscles and subcutaneous tissues. As shown in Figure 5, no significant differences were evident in the expression levels of any of these genes in diabetic vs. nondiabetic muscle and subcutaneous tissues.

## 4. Discussions

In this study, we utilized microarray and RNA-seq expression datasets that were generated using well-characterized pancreatic islets [21] to profile the expression pattern of SARS-CoV-2 host receptors *ACE2*, *ADAM17* and *TMPRSS2* in diabetic and non-diabetic human pancreatic islets. To the best of our knowledge, this is the first study to evaluate the expression of these genes using such a large set of human pancreatic islets. It is well documented that COVID-19 might damage several organs, such as the heart, kidneys, and liver [28], and that abundant SRAS-CoV-2 host receptor’s expression, mainly *ACE2*, in these tissues is crucial for infection [12]. Our study mainly focused on the expression of *ACE2*, *ADAM17*, and *TMPRSS2* genes in human pancreatic islets as such information is still lacking.

Our microarray data revealed a relatively low expression of *ACE2* in human islets. In contrast, Yang et al. have reported abundant expression *ACE2* in human islets using immuno-staining [6]. Moreover, RNA-seq from sorted endocrine cells further confirmed the relatively low expression of *ACE2* in β-cells [27]. Exocrine and α cells expressed comparable levels of *ACE2*. Conversely, *ACE2* expression in mice pancreatic islets was reported mostly in non-β-cells [16]. Although *ADAM17* and *TMPRSS2* are abundant in human islets, RNA-seq from sorted endocrine cells showed that both receptors are highly expressed in exocrine cells as compared to β-cells [27]. While the *ADMA17* expression pattern was similar in human β and α cells, it was mostly restricted to non-β-cells in mice islets [16]. This finding was not surprising as a degree of species specificity between humans and mice has been indicated [29].

Although the dipeptidyl peptidase 4 (*DPP-4*) which serves as a receptor for the MERS-CoV [30,31] may enhance the entry of the virus into pancreatic islets, *ACE2* seems to be the main receptor for SARS-Cov-2 on pancreatic islet cells. This is based on the finding that *ACE2* expression is upregulated in human diabetes/hyperglycemia islets. This is in disagreement with the observation that the expression of *ACE2* and *ADAM17* does not differ between pancreatic islets from db/db mice as compared with non-diabetic controls [16]. However, the expression of *ACE2* and *ADAM17* in the liver, skeletal muscle, and adipose tissue in db/db mice were differentially expressed [16]. Increased expression of *ACE2* in diabetic mice seems to be a cause rather than a consequence as none of the three receptors showed any differential expression in human islets upon short-term exposure hyperglycemia [32]. Hence, it seems that *ACE2*, *ADAM17,* and *TMPRSS2* have a stable expression in pancreatic islets.

Obesity is one of the common risk factors for severe complications and mortality in COVID-19. Obese individuals are difficult to intubate and they experience serious difficulties in breathing due to increased pressure on the diaphragm and chronic inflammation [33]. Epidemiological data reported that obese patients (BMI > 35) are more than seven-fold more likely to be admitted to the ICU as compared with those with BMI < 25 Kg/m^2^ [34]. One explanation of these findings is that COVID-19 has a high affinity for *ACE2* in adipose tissue relative to that of lung tissue [35]. Additionally, the high expression of *ACE2* makes adipose tissue vulnerable to COVID-19 infection. Typically, obese subjects have more adipose tissue and therefore a greater mass of *ACE2*-expressing cells. Although we could not find any effect of BMI on *ACE2* expression in pancreatic islets, the expression of *ADAM17* and *TMPRSS2* was relatively higher in obese donors as compared with non-obese ones.

Increased expression of *ACE2* in females compared to males was unexpected. It is well accepted that males have higher COVID-19-related mortality rates as compared with females, especially in old age [36]. This could be partially explained by the role of sex hormones such as estrogen, and testosterone on the expression of *ACE2* and the pathogenesis of COVID-19. Although our finding is unexplainable, it could be that our female donors were on drugs that caused elevations of *ACE2* receptors such as *ACE*2 inhibitors and angiotensin-receptor blockers (ARBs) [8,37]. It also suggests that increased expression may have a paradoxical protective effect [18,19].

It is also important to acknowledge that several factors might affect gene expression profile in human islets such as donors’ cause of death, the harvest of the organs, preparations of the islets, transportation, and days in cultures.

In conclusion, we introduced the expression profile of SARS-CoV-2 host receptors (*ACE2*, *ADAM17,* and *TMPRSS2*) in human pancreatic islets. Expression of *ACE2*, but not *ADAM17* or *TMPRSS2*, is increased in diabetic human pancreatic islets. The expression of *ADAM17* and *TMPRSS2* was upregulated in obese donors. Further functional and clinical investigations are needed to understand if the *ACE2* receptor in pancreatic islets makes diabetic patients more vulnerable to severe COVID-19 infection-related symptoms compared to non-diabetic patients.

## Figures and Tables

**Table 1:** Clinical characteristics of human islet donors.

	Non-Diabetics	Diabetics
*N* of donors	67	9
Gender (male/female)	37/30	5/4
Age (years)	59 ± 10	60.7 ± 12
Purity %	70 ± 17	60.1 ± 20
BMI (kg/m^2^)	25.9 ± 3.5	28.1 ± 4.5
HbA1c	5.5 ± 1.1	7.1 ± 1.2
Days in culture	3.5 ± 1.9	2 ± 0.9

Data are presented as mean ± SD.

**Table 2:** Expression of *ACE2*, *ADMA17,* and *TMPRSS2* in pancreatic cell type using RNA-seq data from sorted endocrine and exocrine cells.

	β-Cells	α-Cells	Exocrine Cells
*ACE2*	0.27	0.18	0.21
*ADMA17*	1.5	1.4	3.1
*TMPRSS2*	0.55	1.02	5.1

## Abbreviations

SARS-CoV-2	Severe acute respiratory syndrome coronavirus
ACE2	Angiotensin-converting enzyme 2
TMPRSS2	Transmembrane proteases, serine 2
ADAM17	A disintegrin and metalloproteinase 17
BMI	Body mass index
HbA1c	Hemoglobin A2c
T2D	Type 2 diabetes
